# Aspiration Risk and Respiratory Complications in Patients with Esophageal Atresia

**DOI:** 10.3389/fped.2017.00062

**Published:** 2017-04-03

**Authors:** Thomas Kovesi

**Affiliations:** ^1^Children’s Hospital of Eastern Ontario, University of Ottawa, Ottawa, ON, Canada

**Keywords:** esophageal atresia with or without tracheoesophageal fistula, respiratory aspiration, tracheoesophageal fistula, esophageal atresia, gastroesophageal reflux

## Abstract

Chronic, long-term respiratory morbidity (CRM) is common in patients with a history of repaired congenital esophageal atresia, typically associated with tracheoesophageal fistula (EA/TEF). EA/TEF patients are at high risk of having aspiration, and retrospective studies have associated CRM with both recurrent aspiration and atopy. However, studies evaluating the association between CRM in this population and either aspiration or atopy have reported conflicting results. Furthermore, CRM in this population may be due to other related conditions as well, such as tracheomalacia and/or recurrent infections. Aspiration is difficult to confirm, short of lung biopsy. Moreover, even within the largest evidence base assessing the association between CRM and aspiration, which has evaluated the potential relationship between gastroesophageal reflux and asthma, findings are contradictory. Studies attempting to relate CRM to prior aspiration events may inadequately estimate the frequency and severity of previous aspiration episodes. There is convincing evidence documenting that chronic, massive aspiration in patients with repaired EA/TEF is associated with the development of bronchiectasis. While chronic aspiration is likely associated with other CRM in patients with repaired EA/TEF, this does not appear to have been confirmed by the data currently available. Prospective studies that systematically evaluate aspiration risk and allergic disease in patients with repaired EA/TEF and document subsequent CRM will be needed to clarify the causes of CRM in this population. Given the prevalence of CRM, patients with repaired EA/TEF should ideally receive regular follow-up by multidisciplinary teams with expertise in this condition, throughout both childhood and adulthood.

## Introduction

Repaired esophageal atresia, typically associated with congenital tracheoesophageal fistula (EA/TEF), is commonly associated with long-term respiratory morbidity, including recurrent respiratory tract infection, chronic cough, persistently abnormal pulmonary function, and reported asthma (1, 2). There are many potential causes for respiratory complications in this population. Clinically significant tracheomalacia may occur in up to 78% of EA/TEF patients. In infants, tracheomalacia may lead to cyanotic spells (2). In children and adults, tracheomalacia may cause reduced airway clearance, leading to persistent bacterial bronchitis (3, 4). Patients with repaired EA/TEF have multiple, and sometimes interrelated risk factors for aspiration. Aspiration can be due to esophageal dysmotility, which is present in up to 75–100% of EA/TEF patients (2) or esophageal stricture. Gastroesophageal reflux disease (GERD) can also cause lung disease due to aspiration of gastric contents, and 35–58% EA/TEF patients have been reported to experience GERD. Recurrent TEF may arise in about 9% of EA patients. Laryngeal clefts (particularly types 1 and 2) and unilateral or bilateral vocal cord paresis or paralysis appears to be frequent in EA/TEF patients, and often leads to aspiration (5, 6). Vocal cord paresis or paralysis may be, in at least some cases related to EA/TEF corrective surgery (7). Thoracic large vessel malformations such as aberrant right subclavian artery also appear to be abnormally common in EA/TEF patients, and may worsen tracheal and esophageal function, leading to dyspnea, dysphagia, and aspiration (8). Recent studies indicate that other esophageal diseases that impair esophageal function may also be commoner in patients with repaired EA/TEF, further increasing the risk of aspiration, including eosinophilic esophagitis, congenital esophageal stenosis, and heterotopic gastric mucosa in the esophagus (9, 10). Patients with repaired EA/TEF may also develop other respiratory conditions, such as atopy and asthma (2, 11); potentially due to altered gastrointestinal mucosal immunity, they may actually be at increased risk for these conditions. At present, the extent to which chronic respiratory morbidity (CRM) is due to aspiration early in life is unclear. This paper will attempt to summarize current knowledge of the degree to which aspiration is responsible for CRM in these patients.

## Aspiration

Aspiration may be defined as the “inhalation of oral, gastric contents into lower respiratory tract.” Its effect depends on whether the aspirate originates in the pharynx or stomach, whether it is liquid or solid, its pH, the presence of bacteria, and, importantly, its volume and the chronicity of the aspiration. There is evidence that at least 50% healthy adults aspirate small volumes oropharyngeal secretions while asleep, but this is cleared by airway clearance including cough, and by the immune system, leaving no sequelae (12). Aspiration is a general term used to describe a spectrum of acute lung syndromes such as aspiration pneumonitis, aspiration pneumonia, and foreign body aspiration, as well as chronic pathology including diffuse aspiration bronchiolitis, bronchiectasis, organizing pneumonia, and *bronchiolitis obliterans* syndrome (12, 13). Aspiration pneumonitis (Mendelson’s syndrome) is due to regurgitation of gastric contents in the presence of reduced consciousness such as anesthesia, leading to acute lung injury and/or acute respiratory distress syndrome. The aspirated fluid is typically sterile, at least initially, unless the gastric pH had previously been iatrogenically elevated (12). Aspiration pneumonia is caused by the aspiration of infected oropharyngeal secretions in patients who are at risk for aspiration. It is typically a patchy bronchopneumonia that, classically, is in the dependent lobes. Aspiration may lead to necrotizing bronchopneumonia and lung abscess formation (13).

Several research groups have described the pathology and radiology of chronic aspiration. Mukhopadhyay and Katzenstein reported the lung biopsy findings in 59 adults with aspiration pneumonia due to aspiration of particulate matter. Their mean age was 57 years, and, of interest to EA/TEF patients 32% of the cases had esophageal disease or a hiatus hernia. All of the specimens contained alveolar foreign material, including vegetable matter in 92% of cases, and giant cells were present. Eighty-eight cases had cryptogenic organizing pneumonia (*bronchiolitis obliterans* organizing pneumonia) with intraluminal fibroblast plugs in the small bronchioles and alveolar ducts, mainly associated with foreign body-type suppurative granulomas, and foci of bronchopneumonia. A few cases had interstitial foreign material with fibrosis (14). Cardasis et al. reported 25 adult patients with occult aspiration. Their mean age was 62 years. Ninety-six percent had GERD, 32% had a hiatal hernia, and 40% had other esophageal diseases. Biopsies revealed poorly formed granulomas near the bronchioles with evidence of chronic inflammation, and foreign body giant cells and lipoid pneumonia was common. On computerized tomography (CT) imaging, bronchial wall thickening, centrilobular nodules, and tree-in-bud opacities were observed. These were evident mainly in the lower lungs. A few cases had ground glass opacity, interstitial lung disease, or traction bronchiectasis (15). Pereira-Silva described an older patient population, consisting of 13 patients with chronic microaspiration. Their mean age was 71 years. Sixty-nine percent had GERD, 46% had a hiatus hernia, and 23% had esophageal dysfunction. CT scanning demonstrated centrilobular nodules and focal areas of ground glass opacity in all of the patients, and in 85%, these findings were present in the dependent lung regions. Branching (tree-in-bud) opacities were common, and bronchiectasis was evident in 54% (16). It has been reported that on pulmonary function testing (PFT), patients with chronic aspiration commonly have restrictive defects and a low diffusing capacity for carbon monoxide (DLCO) (17).

Aspiration is believed to contribute to a number of chronic lung diseases. Bronchiectasis is believed to be caused by aspiration in 4–18% of patients with non-CF bronchiectasis. El-Serag et al. studied 1,980 neurologically normal children with GERD and 7,920 controls. They reported that the odds ratio (OR) for bronchiectasis was 2.3 (*p* < 0.0001), and pneumonia was 2.3 (*p* < 0.02), among children with GERD (18). By contrast, Piccione et al. reported that in 66 patients with bronchiectasis diagnosed in a specialized Aerodigestive Clinic, aspiration-associated bronchiectasis was strongly associated with severe neurologic impairment. Bronchiectasis was also associated with parental report of GER, but not with the results of esophageal impedance studies or prior fundoplication (19). However, in this population with severe neurologic impairment, CRM may have been predominantly due to chronic aspiration of saliva (20), and the findings may be of less relevance to populations with aspiration predominantly due to other causes. In patients with *bronchiolitis obliterans* syndrome post-lung transplant, GERD appears to play an important role in worsening lung function, and lung function improves with fundoplication (21). In idiopathic pulmonary fibrosis, lung function is possibly worsened by GERD, particularly in scleroderma patients, who may also have esophageal dysfunction. In pulmonary fibrosis patients, there is some evidence that medical anti-reflux therapy may slow the decline in lung function (13).

## Clinical Features of Aspiration

Clinically, the diagnosis of aspiration may be obvious in the case of massive or witnessed choking, but often is under-recognized when due to subclinical microaspiration and misattribution of chronic cough, wheeze, and/or dyspnea.

## Diagnosing Aspiration

Confirming whether aspiration is occurring remains medically challenging. Several tests are available, and they tend to have widely varying reported sensitivity and specificity.

The presence of aspiration is confirmed by lung biopsy showing evidence of a foreign body or foreign body granulomas (*vide supra*), a bronchoscopy demonstrating particulate matter, or when particulate matter is found in the bronchoalveolar lavage (BAL) fluid. Lipid from aspirated food or drinks is ingested by alveolar macrophages, and the presence of lipid-laden macrophages in the BAL has been proposed as evidence of aspiration. The latter may also be quantified as the BAL lipid-laden macrophage index (LLMI). This requires evaluation of 100 macrophages in the BAL, each of which is scored from 0 (no lipid) to 4 (completely opacified). The total is summed and can potentially range from 0 to 400. A value over 100 has been considered as evidence of GERD with aspiration. However, necrosis of alveolar lining cells as a result of severe pneumonia also releases lipid from cell membranes into the BAL. As a result, while the LLMI has “been associated with chronic aspiration” its reported sensitivity varies from 57 to 100%, and its specificity from 57 to 89% (21). Borrelli et al. observed that the LLMI was significantly higher in children with recurrent lung consolidation than in children with asthma (*p* < 0.05), and that the LLMI correlated with the number of reflux and non-acid reflux episodes, and number of episodes reaching the proximal esophagus (*p* < 0.01) on pH-multichannel intraluminal impedance testing. The LLMI also significantly correlated with the number BAL neutrophils (*p* < 0.01) (23). By contrast, Rosen et al. found that in 50 children with a mean age of 6 years, the LLMI was not associated with pH-impedance findings, endoscopic esophagitis, or clinical improvement following fundoplication (24). While BAL pepsin or bile acids have been proposed as markers of aspiration, they require further study (21). Exhaled breath condensate (EBC) was investigated by Fitzpatrick et al. as part of a study of lansoprazole in 110 children with asthma. They found no association between EBC and esophageal pH probe results. Moreover, EBC acidity did not change with lansoprazole and the investigators concluded that EBC does not appear to be useful in the evaluation of the respiratory effects of GERD (25).

Bacterial culture of the BAL fluid may be a surprisingly useful marker of aspiration. Rosen et al. observed that in 46 children with chronic cough or wheezing, with a mean age 74 months, 26% had a positive BAL culture. Cultures grew mainly *Streptococcus pneumoniae* and *Haemophilus influenza*. The presence of a positive BAL culture was predicted by the amount of non-acid reflux or full-column GER on a pH-impedance study, but not a history of pneumonia in previous 6 months. The presence of bacteria in the BAL may also reflect the effectiveness of mucociliary clearance of any aspirated material (26).

In the future, examination of the lower airway microbiome (ribosomal 16 s rRNA ecosystem) may be helpful. The lower airways of healthy people have been reported to have low levels of mainly oral bacteria such as *Prevotella* and *Veillonella* (27), but the microbiome of individuals with chronic aspiration has not been investigated to date.

## Determining the Source of Aspiration

When there is convincing evidence of aspiration, determining its source may be difficult, and different tests may provide conflicting results.

Swallowing dysfunction can be demonstrated by videofluoroscopic swallowing study or, less commonly, fiberoptic endoscopic evaluation. Weir et al. reported that aspiration of thin liquids or post-swallow residue, seen on videofluoroscopic swallowing study, was associated with pneumonia in a broad group of pediatric patients (28). These types of studies have demonstrated that aspiration due to abnormal swallowing is common in EA/TEF patients (29, 30). Esophageal dysfunction can be diagnosed by an upper gastrointestinal (UGI) series or manometry. High-resolution manometry, demonstrating aperistalsis in EA/TEF patients, has been associated with CRM (29). Video manometry, particularly to evaluate a lack of coordination between pharyngeal contraction and relaxation of the upper esophageal sphincter, may also be helpful (31). Recent studies suggesting that laryngeal clefts and vocal cord paresis or paralysis are common in patients with EA/TEF indicate that careful otolaryngologic evaluation of the upper airway should be performed in EA/TEF patients suspected as having aspiration (5, 7). As thoracic vascular malformations may also compromise esophageal function in EA/TEF patients, complete cardiac evaluation of thoracic vessels should also be considered (8). A recurrent or persistent TEF is most often diagnosed by UGI with pull-back study. Many tests are available to diagnose GERD, including UGI, endoscopy, scintigraphy, and impedance/pH probe.

Borrelli et al. used pH-multichannel intraluminal impedance to study 21 children, with a mean age of 4.1 years. They found that 49% of events were non-acid, 74% reached the proximal esophagus, and 80% of the episodes were liquid. The number of reflux episodes, non-acid reflux episodes, and non-acid reflux episodes reaching proximal esophagus were all significantly higher in children with recurrent lung consolidations than in children with asthma (*p* < 0.01) (23). Condino et al. performed these studies in 24 children with asthma and GERD, with a mean age of 33 months. They reported that 51% of events were non-acid. However, there was a low association with symptoms; for example, only 8% of events were associated with cough (32).

Ravelli et al. performed nuclear scintigraphy studies in 51 neurologically normal children with a median age of 6.5 years. GER to the upper 1/3 of the esophagus was detected in 27% of the patients. Delayed gastric emptying (over 90 min) was seen in 53%, and aspiration on a 20-h delayed scan was observed in 49% of children. However, this investigation correlated poorly with other tests. The number of reflux episodes did not differ in children with normal or abnormal pH studies. Seventy-five percent of the children who had aspiration on the delayed scan had a normal pH study, and few of them had histologic esophagitis. Aspiration was associated with CRM, with aspiration seen in 62% of children with recurrent pneumonia and all the infants with apnea. They felt that the sensitivity of the delayed scan was limited by the relatively short half-life of the technetium (33).

## GERD and Asthma

The largest repository of data regarding the association of aspiration with CRM concerns the possible link between GERD and asthma, with over 1,600 studies published. The possible relationship between GERD and asthma is potentially bidirectional, with asthma increasing the risk of GERD, and GERD increasing the severity of asthma. GERD may worsen asthma through a reflex mechanism, with stimulation of vagal nerves in the esophagus by acid, since some of these afferents end in same region of nucleus of the solitary tract where respiratory sensory nerves terminate (34), or through microaspiration, leading to bronchoconstriction and airway inflammation. Non-acid GER may be particularly harmful, as it would likely stimulate airway protective reflexes less. These relationships appear to be complex, with studies showing differing effects of reflux on asthma outcomes. For example, in one study of adults with asthma, omeprazole had no effect on methacholine challenge but did reduce cough sensitivity to capsaicin challenge in the patients who had pH probe-evidence of reflux (35). By contrast, another study observed a correlation between the number of esophageal reflux episodes and airway reactivity as evaluated by methacholine challenge (36).

In a systemic review, Thakkar et al. found that the prevalence of asthma in children with GERD was 13 versus 7% in controls (37). A systemic review in adults noted that the prevalence of GERD, using the Montreal definition, in adults with asthma was 58%, compared to 38% in controls, giving an OR for GERD in adults with asthma of 5.5, and the OR for asthma in patients with GERD was 2.7 (36).

Several studies have evaluated whether GERD is associated with indicators of asthma control. In adults with poor asthma control, abnormal distal or proximal esophageal pH was associated with oral steroid use, and proximal reflux was associated with worse quality of life, but neither was associated with FEV_1_, asthma control, or methacholine challenge (38). By contrast, Kwiecien et al. noted that in 66 children with asthma with a mean age 10 years, night asthma symptoms were associated with a longer time spent at night with an esophageal pH below 4 (39).

Multiple studies have examined whether treating reflux improves asthma outcomes, although the results are contradictory. A study of esomeprazole in 828 adults with asthma and a positive GERD score resulted in very small, but statistically significant improvements in FEV_1_ and quality of life, but not in peak flow, exacerbations, or symptoms (40). In another, study of 207 adults with asthma and GERD symptoms, lansoprazole improved quality of life and reduced exacerbations needing prednisone, but had no effect on lung function or symptoms (41). In a Cochrane systematic review (mainly involving adults), anti-GERD therapy had no consistent effect on peak flows or symptoms (42). Reducing gastric acidity does not appear to be effective in children or adults with asthma and no GERD symptoms (38, 43).

It is important to recognize that proton pump inhibitors do not treat non-acid GER. Rothenberg and Cowles described a series of 235 children with asthma on prednisone who underwent laparoscopic Nissen fundoplication. Ninety percent reduced or stopped their steroids, 90% of children with night symptoms improved, and in the 56 children who underwent PFTs, FEV_1_ improved 26% (44). However, this was a non-controlled, non-blinded study.

In summary, anti-GER therapy appears to improve asthma to some extent in patients with asthma and symptomatic GERD, although the outcomes which improved vary between studies. Some of the variation may be related to differences in how GERD was defined. It is unclear why a treatment effect was seen only in patients with symptomatic GERD.

## Associations with Respiratory Morbidity in Patients with Repaired EA/TEF

Recurrent infection, aspiration, and atopic disease have been associated with CRM in EA/TEF patients. In 68 patients with type C EA/TEF, respiratory complications including recurrent pneumonia were associated with GERD in 74% of patients, with recurrent TEF in 13%, and with esophageal strictures in 10% (45). Recurrent TEF has been associated with cough and with recurrent pneumonia (46). Bronchitis and pneumonia have been associated with dysphagia in 20 children with repaired EA/TEF (47). Shah et al. recently observed that early esophageal stricture formation was associated with recurrent pulmonary infections. GERD was associated with the subsequent performance of an aortopexy to treat severe tracheomalacia (9). A variety of studies have observed that PFT obstructive defects are associated with history of reported GERD (but not results of a 2-h pH probe), as well as choking spells during infancy, and pneumonia during the first 4 years of life (48–50).

Several studies have specifically examined the relationship between GERD and CRM in patients with repaired EA/TEF. However, several of these studies were likely limited by variability in the objective assessment of GERD. In addition, research to date has been retrospective and may underestimate GERD early in life. Peetsold et al. examined the effect of anti-GERD surgery as a marker of past, chronic GERD (51). They reported that neither exercise capacity nor PFT restrictive defects were associated with anti-GERD surgery, but prior surgery was associated with a lower FEV_1_. Using prior anti-GERD surgery as a marker of chronic GERD may be problematic. Anti-GERD surgery is clearly performed because of ongoing, incompletely controlled GERD. However, surgery may prevent GERD from leading to CRM. Furthermore, depending on the degree to which esophageal function is impaired, fundoplication may actually worsen aspiration by causing retention of swallowed material in the esophagus, and oropharyngeal aspiration. Malmström found that in 23 adolescent EA/TEF patients, while 78% had a positive histamine challenge (used as a marker of airway reactivity), airway reactivity was not associated with esophageal symptoms, prior fundoplication, the number of previous reported pneumonias, the results of allergy testing, or physician diagnosis of asthma. Furthermore, airway reticular basement membrane thickening was not associated with gastrointestinal symptoms, esophageal biopsies, atopy, histamine challenge, or exhaled nitric oxide. In addition, PFT restrictive changes were not associated with current esophageal symptoms or past fundoplication (52). Similarly, Legrand et al. found that PFT abnormalities among 57 children with repaired EA/TEF were not associated with GERD symptoms or the results of objective testing, or with prior fundoplication (53). A small study of 26 7-year olds with repaired EA/TEF had similar results, with no association between “esophageal symptoms” and PFT abnormalities, including lung clearance index (as a measure of small airway function). Furthermore, the results of 24-h pH probe were not associated with respiratory symptoms. Olbers et al. also noted that PFT abnormalities in this population could be due to tracheomalacia (54). Pedersen et al. did not find a significant association between abnormalities of esophageal function (determined by endoscopy and pH probe) or a history of recurrent pneumonia, and either obstructive or restrictive PFT defects (55). Sistonen et al. performed histamine challenges in 101 adult patients with repaired EA/TEF. Forty-one percent were positive, and, as expected in individuals with asthma, a positive challenge was associated with atopy or an elevated serum IgE. Unexpectedly, an elevated exhaled nitric oxide was not associated with atopy. While a positive histamine challenge was not associated with esophageal metaplasia on esophageal biopsy, PFT restrictive defects were (56).

Atopic disease, including asthma, may be commoner in EA/TEF patients than in the general population. It is conceivable that chronic aspiration and/or recurrent lower respiratory infection early in life results in persistent airway inflammation and a risk of asthma and other airway diseases later on. In addition to the effects of chronic aspiration, it is possible that altered mucosal immunity in the gastrointestinal tract changes cellular immunity and increases the risk of atopy. Whether asthma in EA/TEF patients is secondary to the effects of aspiration or primary, asthma may cause chronic respiratory symptoms in older patients with EA/TEF. In 334 adult EA/TEF patients, persistent respiratory symptoms were associated with allergies or a family history of allergy (50). In children with EA/TEF and wheezing, 2/3 had a history of atopy (47). Allergies appear to be common in EA/TEF patients. Malmström et al. reported that 15% of children with repaired EA/TEF had allergic rhinitis, and 54% had positive allergy skin tests (52). In adults with repaired EA/TEF, 42% had allergies, 37% had positive allergy tests, and 20% had a high serum IgE. Moreover, these findings were associated with current respiratory symptoms (56). Another smaller study of 28 adults with repaired EA/TEF found that increased airway reactivity, measured using methacholine challenge, was associated with serologic evidence of allergies and with elevated exhaled nitric oxide [generally an indicator of allergic airway inflammation (57)]. However, all of these tests were poorly associated with reported physician-diagnosed asthma (58). By contrast, while Robertson et al. found that methacholine challenge was positive in 48% of 18 EA/TEF patients, it was not associated with symptoms of atopy (59). Similarly, Pedersen et al. did not find that the frequency of allergies (measured by skin prick testing and by serum IgE), abnormal airway reactivity (measured by a mannitol challenge test), or abnormal exhaled nitric oxide differed between EA/TEF patients and a control group being evaluated for GERD (55).

Bronchiectasis is a potentially devastating long-term complication of EA/TEF (22). Using CT scanning, rates of bronchiectasis in EA/TEF survivors may be as high as 27% (4, 55). While neither DeBoer et al. (4) nor Cartabuke et al. (60) examined potential associations with bronchiectasis (59), bronchiectasis in this population has generally been associated with massive aspiration, including patients with gastric or colonic interposition in a selected referral population (22), longstanding GERD (61), massive TEF pouch secretions, trisomy 21 (62), undiagnosed TEF (63), or broncho-esophageal fistula (64, 65).

## Summary

In the general population, bronchiectasis has been clearly associated with GERD and cryptogenic organizing pneumonia can result from chronic aspiration. The relationship between GERD and asthma is unclear, with various studies reporting conflicting asthma morbidities associated with GERD. In patients with repaired EA/TEF, GERD has been inconsistently associated with a low FEV_1_ and with PFT restrictive defects. Early studies have suggested that aspiration early in life in EA/TEF patients is associated with subsequent CRM. However, in more recent studies, while airway reactivity has been at least inconsistently associated with atopy in patients with repaired EA/TEF, increased bronchial hyper-reactivity has not shown to be associated with GERD. Based on a number of case reports and a small series, there is compelling evidence that bronchiectasis in patients with repaired EA/TEF is typically due to massive, chronic aspiration. The lack of consistent evidence that aspiration leads to CRM in patients with repaired EA/TEF almost certainly reflects the variety of ways in which aspiration can be diagnosed, the retrospective nature of research to date, which may well underestimate the severity and chronicity of prior aspiration events, and the effects of previous treatment of aspiration, such as fundoplication. Ideally, prospective studies will be needed carefully documenting esophageal function and GERD, and subsequent CRM. This would likely be the most effective way of quantifying the extent to which aspiration influences subsequent pulmonary morbidity in this population. Research is also required to determine the best methods of diagnosing aspiration, assessing the effects of aspiration on the lower airway microbiome, and assessing whether altered gastrointestinal mucosal immunity affects atopy in these patients. Given the strong association of aspiration with cryptogenic organizing pneumonia and evidence that restrictive pulmonary defects are relatively common in EA/TEF survivors, evaluation by CT and/or biopsy is needed to determine whether at least some of the restrictive impairments seen in patients with repaired EA/TEF are due to cryptogenic organizing pneumonia.

Until additional data are available, there is a compelling need for long-term follow-up of these patients, ideally by multidisciplinary expert teams, both during childhood *and* during adulthood (4, 66). Respiratory follow-up should include serial PFTs including spirometry, measurement of lung volumes, and, possibly, evaluation of bronchodilator responsiveness (67, 68). Chest radiography may be useful (59), and exercise testing and methacholine challenge should be considered when clinically indicated. If bronchiectasis is suspected, it is best diagnosed by CT of the chest. When bronchiectasis is confirmed, urgent evaluation of potential causes of aspiration should be carried out. Based on the evidence currently available, patients with repaired EA/TEF who have CRM should be evaluated for clinically significant tracheomalacia, as well as for aspiration due to swallowing dysfunction, GERD, and/or a recurrent or persistent TEF.

## Author Contributions

TK designed this paper, acquired the data, analyzed and interpreted the data, and gave final approval of the version submitted and any revised version.

## Conflict of Interest Statement

The author declares that the research was conducted in the absence of any commercial or financial relationships that could be construed as a potential conflict of interest. The reviewer YB and handling editor declared their shared affiliation, and the handling editor states that the process nevertheless met the standards of a fair and objective review.
